# Retraction: Ligand assisted Co(ii) catalyzed direct C–H alkylation of aryl ketones with diverse alkyl halides

**DOI:** 10.1039/d6sc90080a

**Published:** 2026-04-13

**Authors:** Aniket Nigade, Saurabh Vinod Parmar, Vidya Avasare

**Affiliations:** a Department of Chemistry, Ashoka University Sonipat Haryana-131029 India vidya.avasare@ashoka.edu.in

## Abstract

Retraction of ‘Ligand assisted Co(ii) catalyzed direct C–H alkylation of aryl ketones with diverse alkyl halides’ by Aniket Nigade *et al.*, *Chem. Sci.*, 2026, https://doi.org/10.1039/d5sc09264g.

The Royal Society of Chemistry, with the agreement of the named authors, hereby wholly retracts this *Chemical Science* article due to concerns with the data, impacting the reliability of the work.

After publication, significant concerns were raised about the validity of the experimental data and an assessment by the journal's editorial board concurred.

Following discussions with the editorial office, the authors state that they have identified inconsistencies in the data provided in the SI.

As the conclusions of the published paper cannot be relied upon, the authors have agreed to retract the paper. The authors sincerely apologize for these errors.

Aniket Nigade, Saurabh Vinod Parmar and Vidya Avasare have agreed to the retraction.

Signed: Aniket Nigade, Saurabh Vinod Parmar and Vidya Avasare, 15^th^ April 2026.

Retraction endorsed by Rebecca Campbell, Executive Editor, *Chemical Science*.

Note added after publication: The text of this retraction has been revised since the initial publication on 13^th^ April 2026.

